# Prolonged third complete remission after busulfan, thiotepa, and autologous stem cell transplant in a primary central nervous system lymphoma patient

**DOI:** 10.1002/ccr3.1630

**Published:** 2018-06-04

**Authors:** Vincent Camus, Sydney Dubois, Stéphane Lepretre, Fabrice Jardin, Hervé Tilly

**Affiliations:** ^1^ Department of Hematology Centre Henri Becquerel Rouen France

**Keywords:** autologous stem cell transplant, busulfan, complete remission, primary central nervous system lymphoma, thiotepa

## Abstract

Primary central nervous system lymphoma (PCNSL) remains a therapeutic challenge due to impaired drugs diffusion as a result of the blood‐brain barrier and high risk of relapse. Patients with good performance status, chemo‐sensitive disease, and eligible for autologous stem cell transplant (ASCT) may benefit from salvage therapy and therapeutic intensification that may allow long‐term remission.

## 1 INTRODUCTION

We present a rare case of primary central nervous system lymphoma (PCNSL) that relapsed twice with a prolonged third complete remission after therapeutic intensification and autologous stem cell transplant. This case opens up the potential beneficial role of busulfan or thiotepa for discussion.

Primary central nervous system lymphoma is a rare but well‐described extranodal high‐grade non‐Hodgkin B‐cell malignancy, accounting for 6.6% of primary brain neoplasms,^1^ with most PCNSLs (>90%) that are diffuse large B‐cell lymphomas (DLBCL). Diagnosis is difficult, based on clusters of clinical, computed tomography (CT), magnetic resonance imaging (MRI), and cerebrospinal fluid (CSF)‐positive cytology arguments. Histological brain biopsy, sometimes very difficult to achieve because of the inaccessibility of the tumor, must always be performed before the implementation of a specific treatment.

Primary central nervous system lymphoma remains a therapeutic challenge because drugs diffusion is down by the blood‐brain barrier, and drugs toxicities reduce the relevance of applicable treatment procedures. We describe here an exceptional case of young PCNSL patient who underwent two relapses and successful salvage chemotherapies with therapeutic intensifications followed by autologous stem cell transplant (ASCT).

## 2 CASE REPORT

A 38‐year‐old male patient, with no relevant medical history, was admitted to the hematology department of the Henri Becquerel Center in Rouen in May 1999 for diplopia with clinical signs of intracranial hypertension. A cerebral CT scan shows left frontal, left parietal, right occipital, and right lateral lesions strongly enhanced after injection of contrast medium with perilesional edema and mass effect on the lateral ventricles. An encephalic MRI is performed which confirms the visible lesions on the initial CT scan. A stereotaxic biopsy is performed and histological samples indicate a large B‐cell lymphoma with a centroblastic phenotype. The initial assessment therefore concludes with the diagnosis of multiple‐site PNCSL of the brain.

The patient presented with a Karnofksy Performance status (KPS) of 70% (ECOG PS = 2), LDH were not elevated, cell blood count and standard chemical test revealed no abnormalities, CSF protein was mildly increased (0.47 g/L) with negative cytology and there were no deep brain lesions. CSF flow cytometry was not performed. The patient’s IELSG prognostic index^2^ was considered “intermediate” and the patient was treated with debulking chemotherapy (cyclophosphamide, vincristine, prednisone) followed by 2 cycles of COPADEM induction (Vincristine 1.4 mg/m² day (D) 1, methotrexate 3000 mg/m² D1, doxorubicin 60 mg/m² D2, cyclophosphamide 250 mg/m²/12 h D2 to D4, methylprednisolone 60 mg/m² D1 to D6 with intrathecal cytarabine injection on D3) followed by 2 cycles of CYM consolidation (methotrexate 3000 mg/m² D1, cytarabine 100 mg/m² D2 to D6, methylprednisolone 60 mg/m² D1 to D6, with intrathecal injection of cytarabine on D3) and whole‐brain radiotherapy with 40 Grays in 16 fractions enabling a first complete remission (CR) lasting 2 years.

First relapse occurred in February 2001 with appearance of gait disorder and micrographia; KPS was still at 70%; the cerebral CT was in favor of a recurrence, with multiple localizations, notably in the basal ganglia (data not shown). These lesions were too deep to biopsy; salvage chemotherapy was decided and the patient was treated with 3 cycles of DIAM, 21 days apart (cytarabine 1500 mg/m² × 2/d on D1‐D2, ifosfamide 1500 mg/m² D1 to D5, dexamethasone 40 mg D1 to D4, methotrexate 3000 mg/m² on D3, with intrathecal injection of methotrexate 15 mg), then therapeutic intensification was conditioned by BEAM‐ARAC high dose (VP 16: 200 mg/m² from D‐7 to D‐4, Cytarabine: 2000 mg/m² infused over 1 hour, ie 3900 mg from D‐7 to D‐4, melphalan: 140 mg/m² or 270 mg at D‐3, dexametasone: 20 mg/day from D‐7 to D‐4) and autologous stem cell transplant (ASCT). Peripheral blood progenitor cells were obtained after 2 cycles of DIAM with 14 × 10^6^/kg CD34(+) cells in the graft with one apheresis collection. No complications occurred during the ASCT and a second CR was obtained.

**Figure 1:**
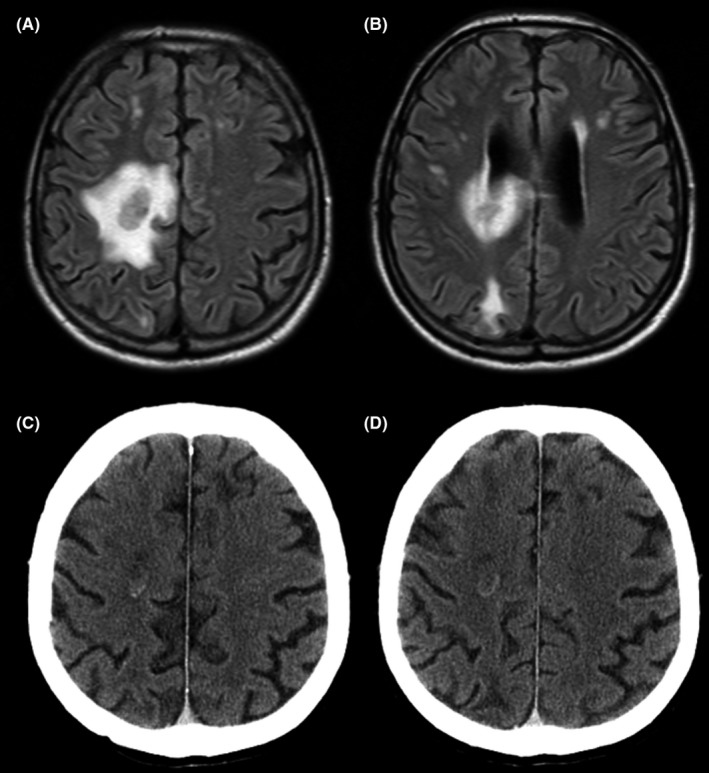
A and B, FLAIR cerebral MRI sequence of October 2007, in favor of a pre‐rolandic right frontal lymphomatous recurrence of 23 × 19 × 15 mm of the supra‐ventricular right white matter with perilesional edema and discrete mass effect on the roof of the right lateral ventricle. C and D, cerebral computed tomography of January 2008, displaying an annular enhancement measuring approximately 19 mm (antero posterior axis) by 17 mm (height) of the right semioval center. A discrete perilesional hypodensity is associated, no mass effect on the adjacent structures is observed, notably there is no abnormality of the ventricular system, which is symmetrical. No evolution is detected after comparison with the MRI of October 2007. The patient did not receive an MRI for cerebral evaluation in 2008 because of the development of claustrophobia contraindicating this procedure

Second relapse was diagnosed in October 2007 with the appearance of a decrease in left visual acuity with uveitis and left‐sided hemiparesis, with KPS at 80%. A brain MRI was performed showing a gadolinium‐enhanced tissue lesion, measured at 23 × 19 × 15 mm, of the right front‐parietal supracentricular white matter with significant perilesional edema, and discreet mass effect on the roof of the right lateral ventricle (Figure 1). The patient initially benefited from a left vitrectomy, which identified a very high concentration of interleukin‐10 (500 IU/L vs normal <10 IU/L). A cerebral stereotactic biopsy of a right prerolandic lesion was performed, confirming the presence of PCNSL. There was no extra‐cerebral involvement. The patient benefited from 4 courses of R‐DIAM (rituximab 375 mg/m² IV at D1 combined with the DIAM chemotherapy previously described), was mobilized by the use of Granulocyte colony‐stimulating factor (GCSF, lenograstim) 34 MUI/d, starting at D12 after the 2nd cycle of R‐DIAM, with apheresis performed at D17 (quantification of hematopoietic progenitors in blood at D17: CD34(+) = 145/μL,) with 9.5 × 10^6^/kg CD34(+) cells in the graft with one apheresis collection. The patient received then a second therapeutic intensification conditioned by thiotepa, busulfan, and cyclophosphamide (thiotepa 250 mg/² on D‐9, D‐8, and D‐7, intravenous busulfan 0.8 mg/kg × 4/on day D‐6, D‐5, and D‐4, cyclophosphamide 60 mg/kg on D‐3 and D‐2) and ASCT. The second ASCT was marked by Grade IV mucositis and *Enterobacter asburiae* and *Pseudomonas aeruginosa* septicemia, which improved over the course of antibiotic therapy. Absolute neutrophil count (ANC) recovery above 0.5 × 10^9^/L and white blood cell (WBC) recovery above 4 × 10^9^/L for 3 consecutive days was achieved at D14 with filgrastim administration from D5 to D14. Platelet count exceeding 20 G/L without transfusion support was obtained at D50.

**Figure 2:**
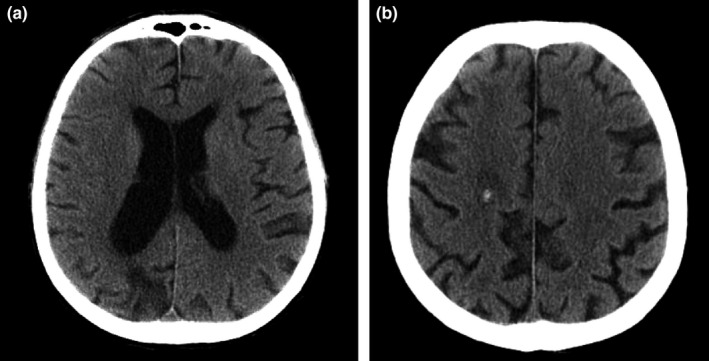
A and B, Cerebral computed tomography of September 2009 identifying the persistence of a discreetly dilated aspect of the lateral ventricles. Persistence of superior right parietal calcification and persistence of right parieto‐occipital cortico‐subcortical hypodensity is also observed, with a sequellar aspect. The aspect is in favor of the persistence of complete remission

A third CR was achieved (Figure 2), and is clinically persisting 9 years after the end of treatment. After this third line treatment, no cognitive disorder was observed, and the patient’s medical follow‐up was marked by:

- Ear, nose, and throat infectious complications with mastoiditis, right rock lysis, and chronic otitis.
- Immunological deficiency with compensated hypogammaglobulinemia.
- Renal failure related to chronic tubulointerstitial nephropathy post‐antibiotherapy, relatively stable since 2010.

## 3 DISCUSSION

This case of PNCSL having relapsed twice with a prolonged third CR is very rare and showcases the potential beneficial role of high‐dose chemotherapy, busulfan, or thiotepa. In the case of our patient, the beneficial role of rituximab could also be suggested, although the patient was able to achieve complete remission with both the DIAM and R‐DIAM regimens. Furthermore, the benefit of the addition of rituximab to high‐dose methotrexate‐based chemotherapy is still unclear, and did not improve PFS in the HOVON study which included 200 patients with newly diagnosed PCNSL.^2^

In addition, our patient also benefited from the strategy of two therapeutic intensifications with ASCT. At the time of first relapse in 2001, thiothepa was not available and BEAM‐adapted conditioning regimen with high‐dose cytarabine followed by ASCT appeared as an interesting therapeutic option that may explain partially the good outcome of the patient, with prolonged second progression‐free survival (PFS2).^3^, ^4^ We also note that our patient has very long time intervals between relapses, well over 1 year, which possibly explains the favorable evolution in the long term. This is consistent with the prognostic factors identified in the cohort of 256 PCNSL published in 2016 by the LOC network^6^: KPS ≥ 70%, sensitivity to first‐line therapy, duration of first CR (>1 year), management at relapse/progression with salvage therapy (vs palliative therapy).

Nevertheless, the results obtained with the second therapeutic intensification with ASCT in terms of better PFS bring the question of the choice of conditioning. The announced central nervous system (CNS) diffusion of busulfan is >80%, while that of cyclophosphamide is 20%‐30%.^7^ Thiotepa is a cytotoxic alkylating agent close to nitrogen mustards, passing the blood‐brain barrier with a ratio of 100% in the CSF, whose efficacy in myeloablative conditioning followed by ASCT in relapsing PNCSL has been well demonstrated since 1990.^5^, ^8^ The excellent CNS entrance of the thiothepa‐busulfan combination contrasts with that of agents in the BEAM‐ARAC high‐dose regimen, where CNS diffusion of BCNU is 15%‐70%, etoposide is <5%, and melphalan is 10%.^7^ It should be noted that the regimen of the second therapeutic intensification of the patient, with thiothepa and busulfan, was quite toxic and marked by numerous infectious complications, which must be taken into account before prescribing this type of conditioning regimen.

Finally, the hypothesis of a combined beneficial effect of thiotepa and busulfan is very likely^9^, ^10^ in the case of this patient.

## 4 CONCLUSION

Primary central nervous system lymphoma is a hematological malignancy that remains chemo‐sensitive at relapse; combinations with molecules such as thiotepa can potentially bring hope for a prolonged CR.

## CONFLICT OF INTEREST

None declared.

## AUTHORS’ CONTRIBUTION

Conception and design: all authors. Administrative support: all authors. Provision of study materials or patients: all authors. Collection and assembly of data: all authors. Data analysis and interpretation: all authors. Manuscript writing: All authors. Final approval of manuscript: All authors.
